# Genome-wide assessment of differential translations with ribosome profiling data

**DOI:** 10.1038/ncomms11194

**Published:** 2016-04-04

**Authors:** Zhengtao Xiao, Qin Zou, Yu Liu, Xuerui Yang

**Affiliations:** 1 MOE Key Laboratory of Bioinformatics, Tsinghua University, Beijing 100084, China; 2 Tsinghua-Peking Joint Center for Life Sciences, Beijing 100084, China; 3 Center for Synthetic and Systems Biology, Tsinghua University, Beijing 100084, China; 4 School of Life Sciences, Tsinghua University, Beijing 100084, China; 5 Joint Graduate Program of Peking–Tsinghua–National Institute of Biological Science, Tsinghua University, Beijing 100084, China

## Abstract

The closely regulated process of mRNA translation is crucial for precise control of protein abundance and quality. Ribosome profiling, a combination of ribosome foot-printing and RNA deep sequencing, has been used in a large variety of studies to quantify genome-wide mRNA translation. Here, we developed Xtail, an analysis pipeline tailored for ribosome profiling data that comprehensively and accurately identifies differentially translated genes in pairwise comparisons. Applied on simulated and real datasets, Xtail exhibits high sensitivity with minimal false-positive rates, outperforming existing methods in the accuracy of quantifying differential translations. With published ribosome profiling datasets, Xtail does not only reveal differentially translated genes that make biological sense, but also uncovers new events of differential translation in human cancer cells on mTOR signalling perturbation and in human primary macrophages on interferon gamma (IFN-γ) treatment. This demonstrates the value of Xtail in providing novel insights into the molecular mechanisms that involve translational dysregulations.

## Abstract (web summary)

The global measurement of ribosome occupancy on mRNAs is commonly used as a proxy in estimating rates of protein synthesis. Here the authors describe Xtail, a computational approach that facilitates the extraction of accurate quantitative insight from ribosome profiling data (Ribo-Seq).

## Graphical abstract

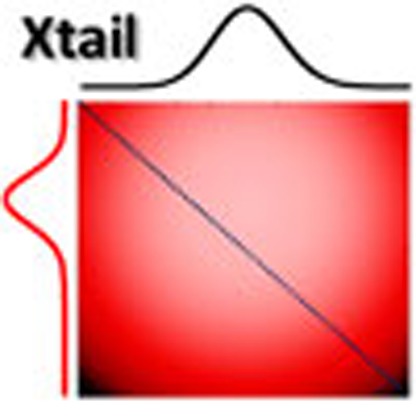

The expression of a protein coding gene involves multiple tightly regulated steps, including DNA transcription, post-transcriptional RNA processing, messenger RNA (mRNA) translation and post-translational processing. Previous research on gene expression regulation has been largely focused on the regulatory levels above translation, such as epigenetic regulations at the DNA and chromatin levels, transcription, RNA processing and decay and so on. However from a global perspective, the abundance of protein—the final product of gene expression—is only partly controlled by transcription or mRNA abundance, and mRNA translation has been increasingly recognized as another major element of gene expression regulation^1^. Indeed, translational dysregulations have been shown to be involved in a large variety of cellular physiological abnormalities, disorders and diseases^2^,^3^,^4^,^5^,^6^.

The global quantitative assessment of mRNA translation has lagged behind the genomic and transcriptomic analyses until recent advances in ribosome profiling, which bring the quantification of translation to the genome-wide level and single-codon resolution^7^. As a combination of ribosome foot-printing and RNA deep sequencing, the procedure of ribosome profiling first generates ribosome-protected mRNA fragments (RPFs, usually around 30 nt) from total mRNA subjected to RNase digestion, and then quantifies RPF abundance with small RNA deep sequencing^8^. The distribution and abundance of RPF reads mapped on a given mRNA transcript reveal the locations and densities of ribosome occupation. Therefore, the number of RPF reads mapped on the coding region of an mRNA species has been frequently used as a measurement of the rate of translation. In parallel, the expression level of each mRNA species in the same sample is also quantified by RNA sequencing to control for the change in RPF abundance that is simply due to altered mRNA copy numbers^8^. Ever since the emergence of ribosome profiling, this powerful technique has been widely applied to study a variety of cellular activities in various organisms and contexts, for example, the adaptation of yeast to amino acid starvation^7^ and oxidative stress^9^, the effects of microRNAs on translation and mRNA decay in zebrafish^4^ and human cells^10^, and the molecular responses of human and mouse cells to proteotoxic stress^11^, heat shock^12^ and perturbations of multiple signalling processes^5^,^13^,^14^. To date, these studies have produced more than 100 ribosome profiling datasets, which are highly valuable resources for understanding translational regulations in a multitude of contexts. Analysis toolsets, tailored for such ribosome profiling data, are therefore badly needed to comprehensively and accurately identify the genes that are subjected to translational dysregulation under specific conditions.

Similar to other high-throughput profiling techniques, ribosome profiling generates genome-wide read-outs, and therefore requires sophisticated statistical tools to screen for true-positive hits from background noise. For a given mRNA species, the abundance of RPF measured by ribosome profiling depends on the translation rate and the mRNA expression level as well. Therefore, a method that integrates both data of RPF and mRNA abundances is needed for isolation and precise quantification of differential translations on top of the transcriptional changes. Last, many of the previous studies using ribosome profiling were performed with very few replicates, therefore necessitating specially designed statistical models that estimate the technical variations and statistical significance properly. Previously in literature, a few analysis strategies have been proposed to search for differential translations with ribosome profiling data, including the quantification of translational efficiency (TE)^7^, anota^15^,^16^, Babel^17^, RiboDiff^18^ and baySeq^19^,^20^. However, most of them have rarely been used in practice with ribosome profiling data. As shown later in the section of results, these methods all suffer, to different extents, from high-false discovery rates and low sensitivities. This indicates that the method strategies and statistical models of these approaches may not be well suited to ribosome profiling data, which bears complex data structure and potentially high levels of noise.

Due in part to the lack of a well-performing analysis method, many of the previous studies with ribosome profiling were focused on individual or a small set of genes undergoing strong differential translation. The advantage of ribosome profiling as a genome-wide assessment of gene translation has not been fully exploited to derive a comprehensive understanding of translational regulations.

Here, we developed a new analysis pipeline, Xtail, to quantify the magnitudes and statistical significances of differential translations at the genome-wide scale with ribosome profiling data. Compared with existing methods, Xtail results in significant improvements in the sensitivity to differential translations and the accuracy of differentiating translational changes from false discoveries, as shown by multiple tests with simulation data and real data from two published studies on mammalian target of rapamycin (mTOR) signalling perturbation in human cancer cells^5^ and interferon gamma (IFN-γ) treatment in human primary macrophages^14^. With these published datasets, Xtail outperforms existing methods in identifying the significant translational dysregulations that make biological sense. Furthermore, by accurately identifying translationally responsive genes and filtering out distracting false-positives, Xtail provides novel biological insights into the molecular and cellular responses to mTOR signalling perturbation in prostate cancer cells. We therefore strongly recommend Xtail for future studies involving ribosome profiling. We also believe that by rediscovering translational dysregulations in a more accurate and comprehensive manner, applications of Xtail on the large collection of published datasets would reveal additional valuable information about the molecular mechanisms of various biological processes.

## Results

### Overview of the Xtail pipeline

As discussed above, for a given mRNA transcript, the abundance of RPF indicates ribosome occupancy on all copies of this mRNA species. The count of RPF reads mapped on the coding region of a given gene depends on both the mRNA abundance and its rate of translation. Therefore, in a comparison between two conditions, differential translation can be characterized by the dissimilarity between the changes in mRNA and RPF expressions across the two conditions. In other words, for a gene undergoing differential translation across two conditions, the fold changes of its RPF and mRNA expression levels would be significantly different. The extent of such difference indicates the magnitude of differential translation. Otherwise, if the changes in RPF and mRNA levels are well coordinated, then the rate of mRNA translation is unaltered. Based on the reasoning above, we designed a method pipeline, Xtail, to quantify the magnitude and statistical significance of the translational change for each gene.

**Figure 1:**
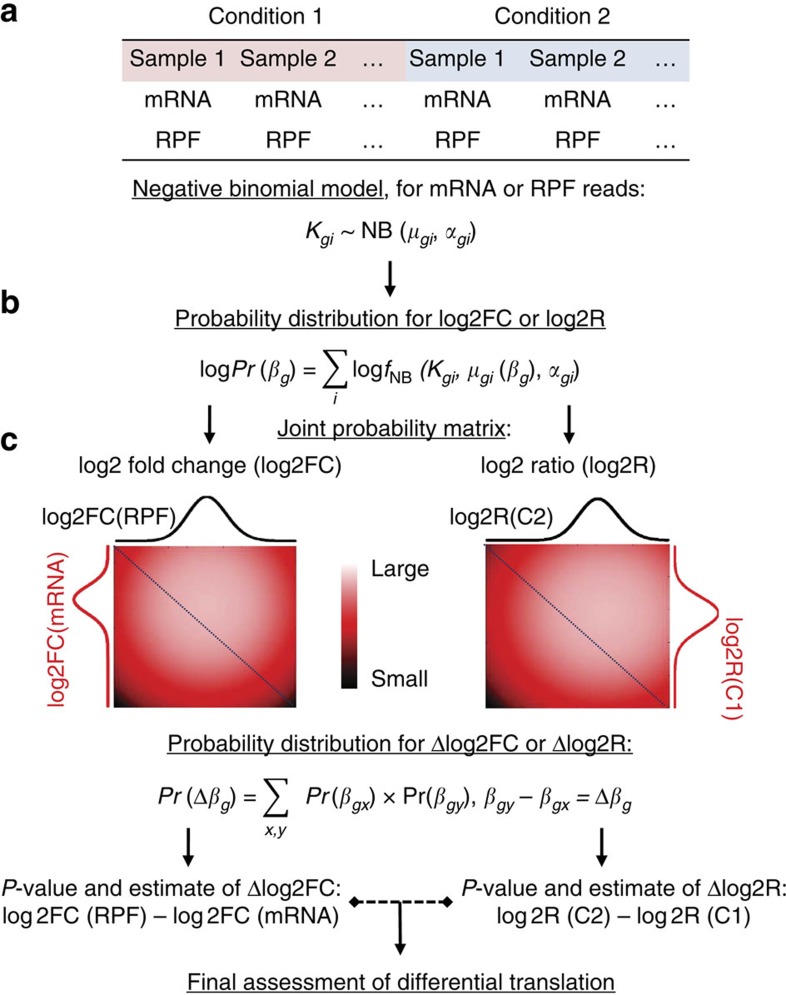
Schematic description of Xtail. Xtail is designed to quantitatively assess gene differential translations across two conditions, C1 and C2, from ribosome profiling data. The analysis process of Xtail includes three major steps: statistical modelling of ribosome profiling data (**a**), establishment of probability distributions for fold changes of mRNA or RPF, or for RPF-to-mRNA ratios (**b**) and evaluation of the statistical significance and magnitude of differential translation for each gene (**c**). Following these steps, two parallel pipelines were implemented to compare the log2 fold changes (log2FC, left) of RPF and mRNA or the log2 ratios (log2R, right) of RPF to mRNA across two conditions. The final assessment of differential translation is derived from one of the two pipelines.

A detailed description of Xtail is given in the method section, and the general outline is illustrated in Fig. 1. First, given that the ribosome profiling is technically on the basis of next-generation sequencing of small RNA libraries, we adopted negative binomial distributions to model the read counts of both mRNA and RPF, similar to many other data analysis methods for RNA sequencing^20^,^21^,^22^,^23^,^24^. We used DESeq2 (refs 21, 22) to estimate the parameters (mean and dispersion) of the negative binomial distributions from normalized read counts.

For each gene, on the basis of the negative binomial distributions of RPF and mRNA read counts, we calculated the posterior probabilities for a range of log2 fold changes (log2FC), and eventually established probability distributions for log2FC of mRNA and RPF. Similarly, in a parallel process, we also derived a probability distribution for log2 ratio of RPF to mRNA (log2R), in each of the two conditions. Next, we generated a joint probability matrix by multiplying the two discrete probability distributions of log2FC (or log2R). Finally, a discrete probability distribution for the differential translation of this particular gene was calculated from this joint probability matrix. The statistical significance and credible interval of this differential translation were then inferred on the basis of this distribution. With this strategy, Xtail quantifies differential translations at the genome-wide level. Please refer to the section of methods for more details.

### Xtail outperforms existing methods with simulated datasets

To compare the performance of Xtail with other existing methods (TE, anota, Babel, RiboDiff and baySeq) in correctly identifying differential translations with ribosome profiling data, we generated a series of simulation datasets with different numbers of samples in each condition (Supplementary Data 2). Different categories of true negatives and true positives of differential translations were predefined and summarized in Supplementary Data 2. The details of data simulation are described in the methods section.

**Figure 2:**
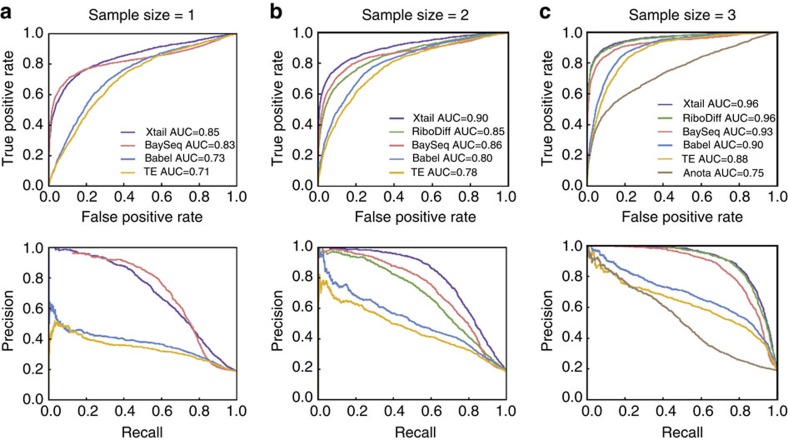
Comparison of Xtail and existing methods with simulated ribosome profiling data. Xtail and other methods were applied on three sets of simulated ribosome profiling data with 1 (**a**), 2 (**b**) or 3 (**c**) samples in each condition. ROC and precision recall curves were prepared to compare the general performances of different methods.

Xtail and other existing methods were applied on the simulated datasets with 1, 2 or 3 samples in each condition. Note that RiboDiff and anota require at least two and three samples per condition, respectively. Receiver operating characteristic (ROC) and precision recall curves were prepared with the results of these methods (Fig. 2a–c). Considering that data simulation involves random selection of genes and assignments of artificial fold changes, we performed the same comparison among these methods with 10 sets of simulation data in total. The areas under the ROC curves from these 10 tests are summarized in Supplementary Fig. 1A–C. From these results, it is clear that among all the methods tested, Xtail obtained the highest sensitivities and the lowest false-positive rates, demonstrating a significant improvement over existing methods. In addition, the performance of Xtail was not too much compromised even in the dataset without replication (Fig. 2a and Supplementary Fig. 1A). This makes Xtail useful in dealing with many of the published ribosome profiling datasets that have no biological replication.

In practice, many biological processes involve gene expression regulations at both levels of transcription and translation. It is well expected to see some genes undergoing transcriptional regulation only and some other transcriptional regulations being reinforced or counteracted by translational regulations. Therefore, it is important to have a method that can precisely extract the effect of translation from a mixture of transcriptional and translational regulations. To specifically test this, we generated another ribosome profiling dataset with the same strategy as above, but granted the same fold changes to the mRNA and RPF of some genes (transcriptional regulation only) and different levels of fold changes to the mRNA and RPF of some other genes (coexisting translational and transcriptional regulations). As shown by the ROC and precision recall curves (Supplementary Fig. 2), Xtail again outperformed the other methods on this dataset, demonstrating its high sensitivity and accuracy in capturing translational regulations on top of altered transcription levels and in excluding transcription-only events.

### Xtail outperforms existing methods with published data

**Figure 3:**
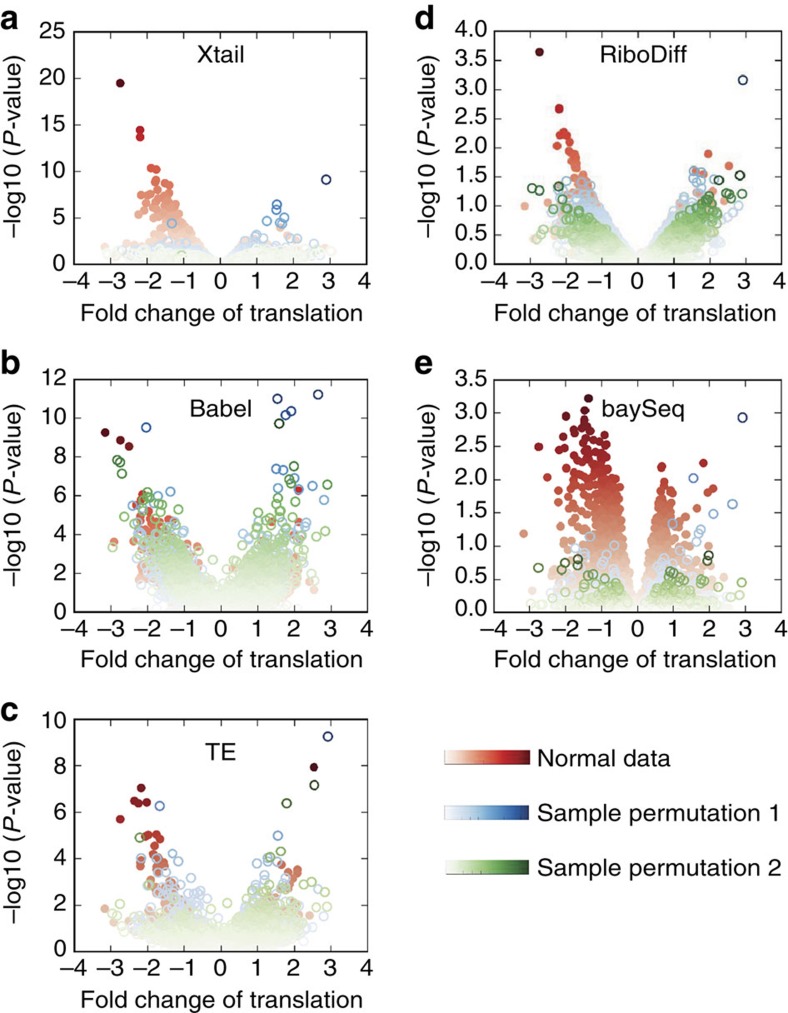
Volcano plots from Xtail and existing methods applied on the PC3 data. Xtail, Babel, TE, RiboDiff and baySeq were applied on the normal and condition-permutated ribosome profiling datasets from the PC3 study. The results are summarized as volcano plots for Xtail (**a**), Babel (**b**), TE (**c**), RiboDiff (**d**) and baySeq (**e**). Log2 of the translational fold change is shown on the horizontal axis, and −log10 of the *P* value is shown on the vertical axis.

**Figure 4:**
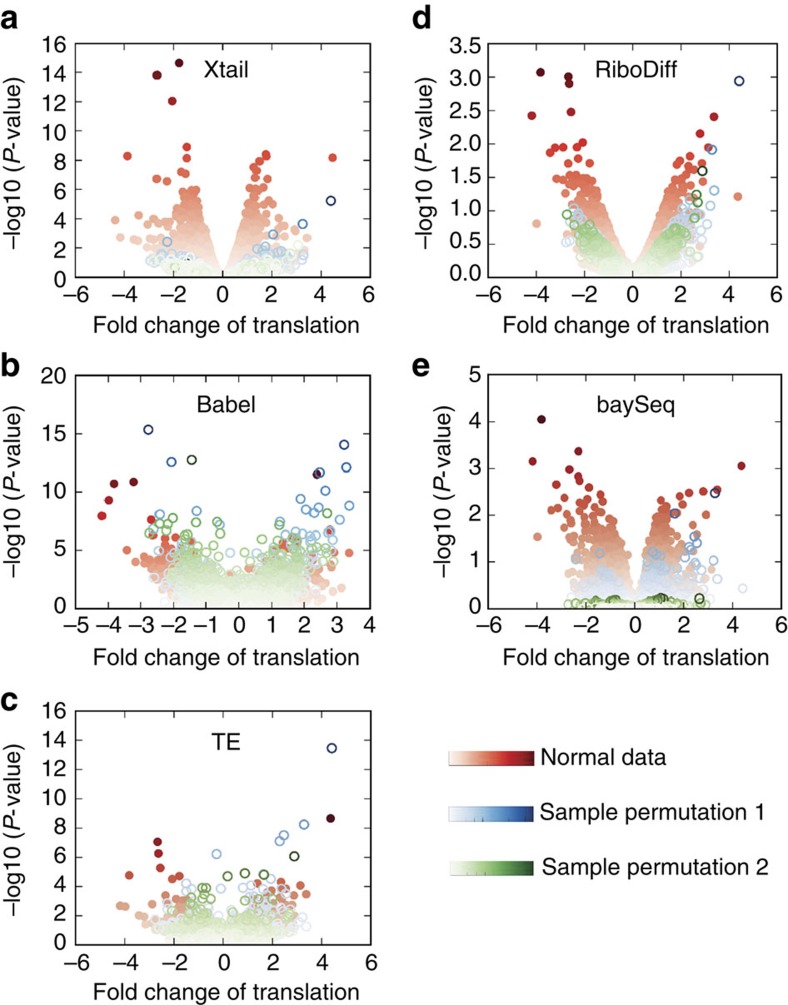
Volcano plots from Xtail and existing methods applied on the macrophage data. Xtail, Babel, TE, RiboDiff and baySeq were applied on the normal and condition-permutated ribosome profiling datasets from the macrophage study. The results are summarized as volcano plots for Xtail (**a**), Babel (**b**), TE (**c**), RiboDiff (**d**) and baySeq (**e**). Log2 of the translational fold change is shown on the horizontal axis, and −log10 of the *P* value is shown on the vertical axis.

To evaluate the performance of Xtail in accurately assessing real differential translations in practice, we used two published ribosome profiling datasets, one from human PC3 cells in response to mTOR signalling perturbation^5^ and the other from human primary macrophages on IFN-γ treatment^14^ (Supplementary Data 3 and 4). These datasets were subjected to the same pre-processing procedure, followed by differential translation analyses with Xtail, Babel, TE, RiboDiff and baySeq. On the basis of the results (Supplementary Data 3 and 4), volcano plots were prepared to illustrate the magnitude (fold change) and the statistical significance of differential translation for each gene (Figs 3 and 4).

In both studies of PC3 cells and macrophages, volcano plots (Figs 3a and 4a) and *P* value distributions (Supplementary Figs 3A and 4A) showed that Xtail nicely differentiated the genes undergoing differential translation from the other majority, much more clearly than the other methods. The distributions of *P* values (−log10) from Xtail have the longest tails (Supplementary Figs 3 and 4), making the statistically significant genes nicely stand out from the majority. These results suggest a high sensitivity of Xtail to differential translations, but do not address concerns about potential false discoveries. According to the results with simulated data (Fig. 2; Supplementary Figs 1 and 2), Xtail has the lowest false discovery rate. However, to further address this issue, we shuffled the experimental conditions in these two datasets, by swapping one of the two controls with either of the two treatments. Two permutated datasets from either the PC3 or macrophage studies were generated, which in theory should not yield any significant differential translation. Indeed, as shown in Figs 3a and 4a, with Xtail, sample shuffling eliminated almost all the significant results from the original unshuffled data, in both PC3 and macrophage datasets (Supplementary Data 5 and 6). The *P* value distributions from these two permutated datasets are much tighter than the ones from the normal dataset, and the long tails are almost completely eliminated after sample permutations (Supplementary Figs 3A and 4A). By contrast, with the other methods, condition-permutated comparisons yielded large numbers of differential translations, most of which should be false discoveries (Figs 3b–e and 4b–e). Their *P* value distributions are in the same ranges as the ones from the normal comparisons of control versus treatment (Supplementary Figs 3 and 4), suggesting high-false discovery rates. The performance of baySeq in this test appears better than Babel, TE and RiboDiff, but still not as good as Xtail (Figs 3 and 4; Supplementary Figs 3,4).

To further compare the results from these five methods, we examined the mRNA and RPF expression levels of the top 100 differentially translated genes identified by these methods (Supplementary Data 3 and 4). From both the PC3 (Supplementary Fig. 5A) and macrophage (Supplementary Fig. 5B) data, the top 100 differentially translated genes reported by Xtail generally have mid- to high-expression levels of mRNA or RPF (normalized read counts above 100). However, with the other methods, various numbers of the top100 genes fall in the low-expression range (normalized read counts far below 100). In practice, low-expression genes with read counts far fewer than 100 bear too much technical variance, usually leading to unreliable estimations of the expression levels. This issue raises serious concerns about potential false discoveries among these low-expression genes due to the bias of their expression estimation.

In summary, as shown by multiple tests with the two published datasets, Xtail exhibited superior sensitivities and specificities to the other existing methods. Next, for further comparison, we examined the biological relevance of the results from all these five methods.

### Biological relevance of the results

**Figure 5:**
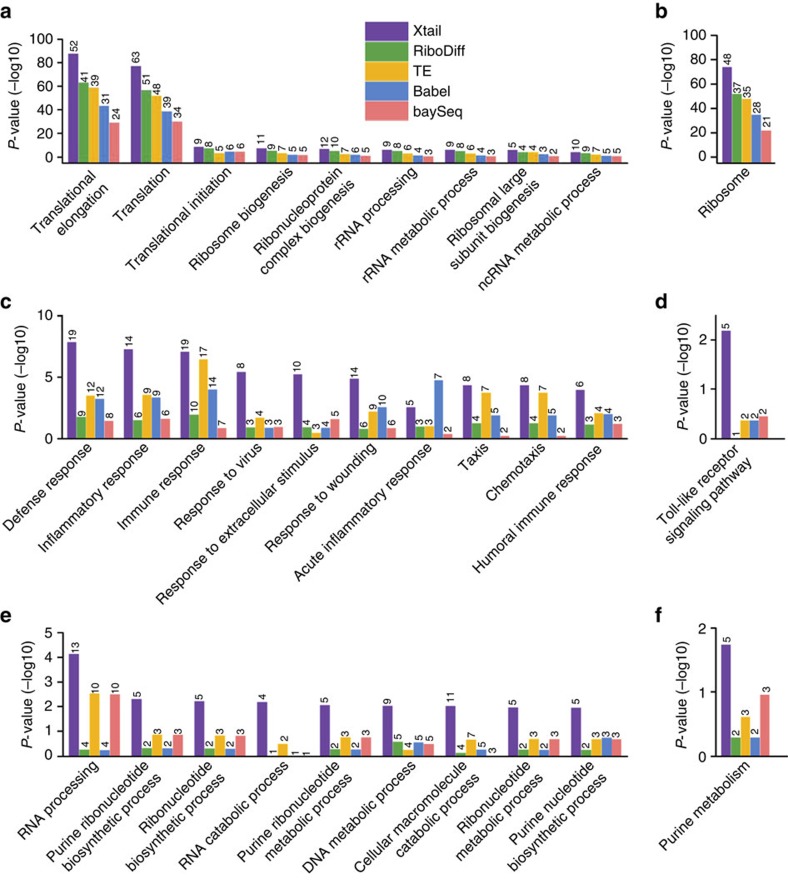
Biological processes and functional pathways enriched in the differentially translated genes identified by different methods. (**a**,**b**) Biological processes in GO (**a**) and KEGG pathways (**b**) enriched in top 100 down-regulated genes identified by five methods with the PC3 data. (**c**–**f**) Biological processes (**c**,**e**) and KEGG pathways (**d**,**f**) enriched in top 100 upregulated (**c**,**d**) and down-regulated (**e**,**f**) genes identified by five methods with the macrophage data. *P* values of the enrichments are shown on the vertical axes in −log10 scale. The number on top of each bar represents the number of genes, within the up- or down-regulated gene sets, falling in the specified category of biological process or pathway.

As shown by the volcano plot in Fig. 3a, upon PP242 treatment on PC3 cells, significant differential translations identified by Xtail are almost exclusively down-regulated, which is in good agreement with the experimental condition of PP242, a potent mTOR inhibitor^5^. However, Babel, TE and RiboDiff failed to recapitulate such pattern (Fig. 3b–d), suggesting low sensitivities and/or high-false discovery rates of these two methods. Specifically, Xtail identified 109 differentially translated genes with false discovery rate (FDR)<0.1, and among these genes, only two are up-regulated (ranked 70th and 104th by *P* value). We selected the top 100 translationally down-regulated genes (with *P* values from 3.3E−20 to 6.5E−4), and performed Gene Ontology (GO) and the KEGG pathway enrichment analyses to identify the biological functions and processes that were perturbed due to the gene translation repression by PP242 (Fig. 5a,b). In addition, the same analyses were performed with the top 100 down-regulated genes identified by the other four methods as well (Fig. 5a,b). For each method, these genes should be considered significant as they are far apart from the majority in the *P* value distribution (Supplementary Fig. 3).

In general, on the basis of the results of Xtail, the biological processes enriched in the top translationally down-regulated genes by PP242 treatment are almost all about translation processes, ribosome biogenesis and RNA processing (Fig. 5a,b), which is well expected. However, although these processes are also enriched in the results from the other methods, their levels of enrichments are much lower than those from Xtail (Fig. 5a,b).

As shown by the volcano plot in Fig. 4a, IFN-γ treatment in primary macrophages results in translational up- or down-regulations of many genes. It has been well characterized that type I and II interferons induce transcriptional up-regulations of interferon stimulated genes (ISGs) through the JAK–STAT pathway^25^. IFN-γ is well known for triggering activation of macrophages by up-regulation of ISGs as discussed above. However, the complex effects of IFN-γ on macrophages cannot be fully explained by transcriptional responses alone. Indeed, IFN-γ was also found to prime macrophages via crosstalks with the multiple signalling pathways, leading to substantially altered cell states^26^. Multiple studies have shown that IFN-γ activates the PI3K–AKT–mTOR pathway^27^,^28^, resulting in elevated translation of ISGs^29^,^30^. Evidence also suggested that the altered protein levels of some ISGs in response to IFNs are actually due to the mRNA translational regulations rather than transcription^29^. In addition to the mTOR pathway, the MEK/ERK/MNK pathway activated by IFN-γ also contributes to the alteration of translation programs by IFN-γ^29^. On the other hand, IFN-γ treatment was also shown to induce translation repression for many other mRNA species by activating the IFN-γ-activated inhibitor of translation (GAIT) complex^31^.

Xtail identified 286 (146 translationally up-regulated and 140 down-regulated) genes with FDR<0.1. We chose the top 100 translationally up- and down-regulated genes separately, and performed GO and the KEGG pathway enrichment analyses to assess potential functions or biological processes enriched in these up- and down-regulation gene sets (Fig. 5c–f). Similarly, the same number of translationally up- and down-regulated genes identified by the other four methods were also tested for GO and KEGG enrichments. The *P* value cut-offs used to select these genes are shown in Supplementary Fig. 4.

In general, the biological processes and pathways enriched in the translationally up-regulated genes include immune response, inflammatory response, responses to virus and other stimulus, Toll-like receptor signalling pathway, chemotaxis and so on (Fig. 5c,d). This is in good agreement with previous knowledge about cellular responses to IFN-γ as an immune interferon, a cytokine and a chemoattractant. This is also consistent with the previous finding, as discussed above, that IFN-γ induces translational up-regulation of ISGs, in addition to its effects on ISGs transcription.

RNA processing and nucleotide metabolism are enriched in the list of down-regulated genes from Xtail (Fig. 5e,f). The original article, in which the macrophage ribosome profiling data was generated, nicely demonstrated that IFN-γ is indeed involved in reprograming macrophage metabolism^14^. It is unclear how IFN-γ also participates in regulating RNA processing. However, given the antiviral function of IFN-γ, it is plausible to hypothesize that IFN-γ may suppress or perturb virus and host RNA processing by inhibiting the translation of certain genes involved in RNA processing. As discussed above, IFN-γ induces translational repression of some genes by activating the GAIT complex. A subset of ISGs have also been shown to repress translations of some mRNA species^32^. Thus, it is worth further investigation to see whether and how IFN-γ regulates RNA processing, which is potentially mediated by the translation-repressing functions of some ISGs and the GAIT complex.

The performances of the other four methods in this test are not consistent (Fig. 5c–f). Their results also showed enrichments of some biological processes discussed above, but at much lower levels (Fig. 5c–f). Taken together, the functional enrichment analyses above demonstrated Xtail's superior performance in obtaining results of high biological relevance. Yet this may raise a concern about Xtail's specificity in capturing real translational regulations and excluding the genes that are only subjected to transcriptional regulation, especially in the IFN-γ study. Indeed, if a method failed to differentiate between translational and transcriptional regulations and captured genes that are altered at either of the two levels, then the functional enrichments in its results may appear better than in other methods, which specifically identify translational regulations only. However, this is not the case for Xtail for the following reasons. (1) As discussed earlier with simulation data, in which genes undergoing transcriptional regulation only, and genes subjected to both translational and transcriptional regulations were predefined, Xtail exhibited the highest sensitivity and accuracy in capturing translational regulations on top of the altered transcription levels and in excluding the transcription-only events (Supplementary Fig. 2). (2) In Supplementary Figs 6 and 7, we reproduced the volcano plots in Figs 3 and 4 (without sample shuffling), but color-coded each dot with the *P* value of the mRNA differential expression obtained with DEseq2 (ref. 22). In the results of Xtail from the PC3 and macrophage datasets (Supplementary Figs 6A and 7A), most of the significant differential translations are indeed translational regulation only, although there are some events of combined transcriptional and translational regulations. These figures also showed that Xtail does not favour transcriptionally regulated genes more than any other methods (Supplementary Figs 6 and 7). Therefore, the better enrichments of the GO and KEGG terms in Xtail's results should be due to Xtail's higher efficiency and accuracy in identifying real translational regulations.

As discussed above, Xtail exhibited high sensitivity and exceptionally low-false discovery rate, which ensured its efficiency and precision in identifying true differential translation events. Its results make sense of the biological processes that are perturbed by the experimental conditions. We therefore believe that Xtail is of great value as a powerful method, not only for future studies with ribosome profiling, but also for reanalysis of published data to gain novel biological information. To exemplify this, we examined the results of the differential translation analysis with the PC3 data, to pursue novel biological insights into the translational dysregulations involved in mTOR inhibition by PP242 in prostate cancer cells.

### New biological insights from the results of Xtail

In the original study that produced the ribosome profiling data, PP242 was used as an mTOR inhibitor, which suppressed PC3 cell migration and prostate cancer metastasis^5^. We examined the top 20 differentially translated genes identified by Xtail, which are all down-regulationed upon PP242 treatment in PC3 cells (Supplementary Data 7). Thirteen of these genes are ribosomal proteins, translation initiation and elongation factors. Indeed, it has long been known that the inhibition of mTOR signalling suppresses the translation of translation elongation factors^33^ and a large group of ribosomal proteins, *in vivo* and *in vitro*^34^,^35^. The translations of some translation initiation factors, including EIF4B and EIF3F, in this top 20 list were also found to be sensitive to mTOR inhibition^36^,^37^. Three of the other seven genes were also among the top-ranked genes identified by other methods, whereas the other four genes (*GNB2L1*, *VIM*, *IPO7* and *AHCY*) were not well supported by methods other than Xtail. We therefore focused on these four genes in the following discussion.

GNB2L1, also called RACK1, is a well-studied scaffold protein that regulates multiple signalling pathways involved in critical cellular processes, such as mRNA translation, cell motility and survival^38^. Serving as an integral ribosomal protein, GNB2L1 binds to the 40S ribosomal subunit and directly contacts with ribosomal RNA (rRNA)^39^. It recruits activated protein kinase C (PKC), which phosphorylates eIF6, resulting in the release of eIF6, the assembly of 80S ribosome and the activation of translation^40^. It has been shown that the translation of GNB2L1 is subjected to inhibition by rapamycin, another mTOR signalling inhibitor^41^. On the basis of above evidence, it is well expected to see GNB2L1 among the top translationally responsive genes on PP242 treatment. Furthermore, GNB2L1 is also well known for its function in cancer as a key adaptor protein involved in multiple cancer-related pathways or interactions^38^. One of the key functions of GNB2L1 is the regulation of cell adhesion, migration, invasion and eventually the promotion of metastasis. This function is attributed, at least partially, to the intermolecular complex composed of GNB2L1, vimentin (VIM) and FAK, in which GNB2L1 is required for stabilization of the complex^42^. Interestingly, VIM was also identified by Xtail as one of the top translationally suppressed genes by PP242 treatment, which has been experimentally validated by the original study in which the PC3 data was generated^5^. Therefore, from the top 20 responsive gene list, a well-supported hypothesis was generated that the inhibitory effect of PP242 on PC3 cell metastasis is mediated, in part, by the translational down-regulation of GNB2L1 and VIM. This has not been studied before, and this valuable information is not immediately apparent in the results of TE or Babel.

IPO7, also called RANBP7, is one of the importin β-like transport receptors, whose major function is in nuclear protein import^43^. Interestingly, IPO7 has been shown to directly bind and import ribosomal proteins into mammalian cell nuclei^44^. Indeed, during ribosome biogenesis, ribosomal proteins were first imported into the nucleolus and assembled with rRNA before being exported^45^. The expression regulation of IPO7 at the translation level has not been well studied. The identification of IPO7 by Xtail suggests that mTOR inhibition does not only suppress translation via the initiation and elongation factors, but may also disturb ribosome biogenesis by inhibiting ribosomal protein import.

AHCY, also called SAHH, belongs to the adenosylhomocysteinase family, which catalyses the hydrolysis of *S*-adenosylhomocysteine in methylation reactions^46^. Few studies have investigated the regulation of AHCY expression and its involvement in cancer. However, a study showed that AHCY is involved in promoting mRNA cap methylation, which is essential for mRNA translation^47^. This function of AHCY mediates c-Myc-induced mRNA cap methylation, protein synthesis and cell proliferation. Another study showed that the inhibition of AHCY suppressed tumour cell proliferation and tumour growth *in vivo*^48^. On the basis of the literature survey above and the identification of AHCY as one of the top responders of mTOR inhibition, it is a plausible hypothesis that the inhibition of mTOR suppresses mRNA translation partly by translationally down-regulating AHCY, which is required for mRNA cap methylation. It is also worth further investigation to see whether the translational inhibition of AHCY could be another way of suppressing tumour cell growth by inhibiting mRNA cap methylation and translation.

In summary, a quick survey of the top 20 results of Xtail provided valuable insights into the molecular responses of PC3 cells to mTOR inhibition, and generated novel but plausible hypotheses about the molecular mechanism of PP242-supressed tumour growth and metastasis. These results reaffirm the outstanding performance of Xtail in precisely, and comprehensively identifying events of translational dysregulation. This example also showed the potential of Xtail in reanalyzing published ribosome profiling datasets for more comprehensive and novel understandings of translational regulations in various contexts.

## Discussion

mRNA translation is being increasingly recognized as a crucial step in the extensively regulated process of gene expression. Ribosome profiling has become a standard method to quantify the rates of mRNA translations and to identify irregular translational processes on a genome-wide scale. Until now, a large number of datasets have been generated by ribosome profiling, shaping the landscapes of translational regulation in a variety of experimental and physiological conditions. These studies have greatly improved our understanding of translational controls in cellular physiology and various diseases. However, many of the previous works focused on small sets of genes that showed the strongest signal of translational dysregulations or were of particular interest to the investigators. This is due partly to the lack of a well-performing method that can comprehensively and precisely extract differential translations from ribosome profiling data.

Previously, several methods have been proposed for the differential translation analysis with ribosome profiling data. Among them, Babel uses an errors-in-variables regression model to compare ribosome associations within and between conditions^17^. TE simply transforms translational fold changes into *Z*-scores by first grouping genes according to their expression levels and then fitting a normal distribution within each group^49^. baySeq was designed for the differential expression analysis with RNA-seq data^19^,^20^. Here the analysis procedure in baySeq for paired samples was used for differential translation analysis. RiboDiff takes the mRNA abundance variability as a confounding factor and estimates the differential translation efficiency by comparing the RPF abundances^18^. Anota uses the analysis of partial variance to weigh the contribution of differential mRNA expression to the observed changes in RPF levels^15^,^16^. Although most of these methods have been available for a long time, they are far from popular in the published studies with ribosome profiling. Our analyses showed that these existing methods suffer from high-false discoveries and low sensitivities, to different extents in various tests. Therefore, due to the complicated structure and relatively high noise levels of ribosome profiling data, a more sophisticatedly designed and well-performing method is urgently needed.

In the present study, we developed Xtail, an integrative analysis pipeline, to systematically assess differential translations with ribosome profiling data. Xtail applies two parallel pipelines to quantify the discoordination between the mRNA and RPF changes or between the RPF-to-mRNA ratios in two conditions, as a measurement of differential translation. As shown by the tests performed on both simulated and real data, Xtail achieved very high sensitivity while keeping the type I error minimal, demonstrating a substantial improvement over the existing methods. When applied on published datasets from mTOR signalling perturbation in cancer cells and IFN-γ treatment in primary macrophages, Xtail revealed translationally responsive genes that make biological sense and more importantly, provide novel insights into the molecular machineries of these biological processes. This nicely illustrates the value of Xtail, not only in processing future ribosome profiling data, but also in revisiting previously published studies. We propose that reanalyzing the large collections of previously published ribosome profiling data with Xtail would generate more complete and accurate surveys of translational dysregulations in various experimental and physiological contexts. These surveys would provide unique mechanistic information, at the level of genome-wide translational regulation, for better understanding of important biological processes, such as stress responses, key pathway perturbations and disease onset.

## Methods

### Pre-processing of raw data

The initial processing (for example, adaptor removal, QC, selection of reads and so on), mapping and counting of raw sequencing reads are not in the scope of Xtail. Different strategies of data pre-processing for ribosome profiling have been used in literature^3^,^8^,^19^,^50^,^51^,^52^. In the present study, files of raw reads (Sequence Read Archive, or SRA format) were downloaded from the Gene Expression Omnibus (GEO) database, and the SRA Toolkit was used to convert SRA to the FASTQ format. The cutadapt program^53^ was used to trim the 3′ adaptor in the raw reads of both mRNA and RPF. Low quality reads with Phred quality score >20 (>50% of bases) were removed using the fastx quality filter (http://hannonlab.cshl.edu/fastx_toolkit/). Next, sequencing reads originating from rRNAs were identified and discarded by aligning the reads to rRNA sequences of the particular species using Bowtie (version 1.1.2) with no mismatch allowed. The remaining reads were then mapped to the genome and spliced transcripts using Tophat2 v2.1.0 with default parameters except for the following: –bowtie1, –no-novel-juncs, –read-realign-edit-dist=0. Two mismatches were allowed for this step of alignment.

For each gene, mRNA expression was estimated by mRNA-seq reads, which were counted using HTSeq-count^54^ in intersection-strict mode. For quantification of RPF, multiple filters were implemented on raw reads to reduce the technical noise of ribosome profiling, and extract the reads originating from ribosome-binding and translating sequences in coding regions. First, RPF reads with length between 26 and 34 nt were deemed high quality and most likely to be from ribosome occupation in mammalian cells^51^,^55^. It has been shown that reads around this range exhibit obvious 3-bp periodicity, suggesting high enrichment of ribosome-binding sequences^7^,^52^. Second, to reduce noise due to multiple alignments, only the reads uniquely mapped to the coding regions were counted for RPF. Third, due to the potential accumulation of ribosomes around the starts and ends of coding regions^3^,^55^, reads aligned to the first 15 and last 5 codons were excluded for the counting of RPF. RPF reads passing all the filters above were counted using a custom script written in Python (Supplementary Data 1).

After the pre-processing procedure described above, Xtail takes in raw read counts of RPF and mRNA, and performs median-of-ratios normalization^56^ by default. Alternatively, users can choose to provide normalized read counts and skip the built-in normalization in Xtail.

### Workflow of Xtail

Xtail is based on a simple assumption that under certain experimental or physiological condition, if a gene was not subjected to any translational dysregulation, the abundance of RPF from its coding region, as revealed by ribosome profiling, would either remain undisturbed if the mRNA expression remains stable or change coordinately with the mRNA expression. In other words, if the response of RPF abundance to the experimental condition was not coordinated with that of mRNA expression, then it is highly suspected that this gene undergoes translational dysregulation. Therefore, Xtail first estimates the fold changes of RPF and mRNA across two conditions separately, and then uses the ratio of these twofold changes (ratio of fold changes) to quantify the magnitude of differential translation. Mathematically, this is equivalent to first estimating the ratios of RPF to mRNA in two conditions, and then taking the fold change of these two ratios (fold change of ratios) across two conditions.

As illustrated in Fig. 1, the analysis pipeline of Xtail consists of the following three major steps: statistical modelling of ribosome profiling data (Fig. 1a), establishment of probability distributions for fold changes of mRNA and RPF or for RPF-to-mRNA ratios (Fig. 1b) and evaluation of the statistical significance and magnitude of the differential translation for each gene (Fig. 1c).

#### Statistical modelling of ribosome profiling data

Ribosome profiling is based on small RNA sequencing technique. For RNA sequencing with very limited sample numbers, the Negative Binomial (NB) model has been widely used to estimate the distributions of read counts across samples. Compared to Poisson distribution, NB is more flexible, and it allows technical or biological variability that may lead to a variance higher than the mean. NB models have been widely used and shown to work well in multiple analysis methods for RNA sequencing data, such as DESeq^21^, DESeq2 (ref. 22), edgeR^23^, baySeq^20^ and sSeq^24^. Therefore, we adopted the same strategy and assumed NB distributions for normalized read counts of mRNA and RPF in Xtail. The classical problem of estimating the dispersions *α* and means *μ* of these NB distributions has been well addressed by a number of data-processing tools sophisticatedly designed for RNA sequencing, including DSS^57^, edgeR^23^ and DESeq2 (ref. 22). Xtail relies on DEseq2 to establish NB models and estimate the dispersions *α* and means *μ*.

Specifically, for each gene, normalized read counts of RPF or mRNA in all samples were used to fit NB distributions with dispersions *α* and means *μ*. mRNA or RPF read count *K* for gene *g* in sample *i* is described as:


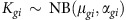


As mentioned earlier, the raw read counts were then scaled by a normalization factor (*s*_*i*_) using the median-of-ratios normalization method as described and used in multiple algorithms including DESeq^21^, DESeq2 (ref. 22) and DEXSeq^56^:


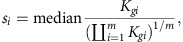


where *m* is the total number of samples. Many approaches have been used previously in literature to estimate the parameters of NB distributions^20^,^23^,^57^. Here we employed DESeq2 to estimate the posterior mean and dispersion of mRNA or RPF, separately, for each gene. This process includes the “Empirical Bayes shrinkage” method in DESeq2 (ref. 22) to control the bias of dispersion estimation due to biological or technical variability. This method is based on the assumption that genes with similar expression levels also share similar levels of dispersion. Specifically, the dispersions of each gene were first used to fit a smoothed curve, and then shrunk toward the curve to obtain the updated values of dispersion. Please refer to DESeq2 (ref. 22) for a more detailed description of this process.

#### Establishment of probability distributions for fold changes of mRNA and RPF or for RPF-to-mRNA ratios

As discussed above, Xtail defines the translational change across two conditions as the difference between the log2FC of RPF and mRNA, or between the log2 ratios of RPF to mRNA (log2R) in the two conditions. Therefore, to establish a probability distribution for the translational changes, which would be used to infer statistical significance of differential translations, we need to estimate the distributions for log2FC of RPF and mRNA or for log2R in two conditions.

First, the mean of mRNA or RPF expression in each sample, *μ*_*gi*_, can be written with the following generalized linear model:


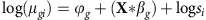


where intercept *ϕ*_*g*_ is the expression base level, **X** is a 0/1 vector of covariates indicating whether sample *i* belongs to condition one or two, and coefficient *β*_*g*_ represents the log fold change of expression compared to the base level *ϕ*_*g*_. For the RPF or mRNA of each gene, on the basis of the NB distribution of its read count, the posterior probability for a given value of *β*_*g*_ can be calculated by


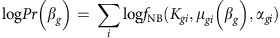


where *f*_NB_ is the probability mass function of the NB distribution, which is given by


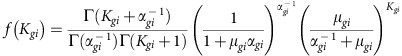


On the basis of the definition above, Xtail derives a discrete probability distribution of *β*_*g*_ (log2FC), for either mRNA or RPF, by calculating the probabilities *Pr*(*β*_*g*_) for 10,000 values of *β*_*g*_, which are uniformly distributed in the range of [*β*_*g*,L_, *β*_*g*,U_]. *β*_*g*,L_ and *β*_*g*,U_, the lower and upper limits of the sampling range, were located where *Pr*(*β*_*g*_) reaches 1e−50 or below. Using the same strategy, Xtail also derives a discrete probability distribution of log2R in each of the two conditions. Here *ϕ*_*g*_ was defined as mRNA expression, and *β*_*g*_ represents the log2 RPF-to-mRNA ratio.

#### Evaluation of the statistical significance and magnitude of the differential translation for each gene

Finally, Xtail tests for each gene whether there is a significant difference between log2FC of RPF and log2FC of mRNA (or between log2R values in two conditions).

As Fig. 1 shows, in one of the two parallel analysis pipelines, Xtail generates a joint probability matrix, which is the outer product of the two probability density distributions for log2FC of mRNA and log2FC of RPF. By definition, the sum of the upper triangle is the posterior probability of the translational fold change being >1 (log2FC of RPF greater than log2FC of mRNA), and the sum of the lower triangle is the probability of the fold change being smaller than 1. If the translational fold change tended to be greater than 1 (with a probability larger than 0.5), we calculated the posterior probability of the fold change being 1 or even more extreme (smaller than 1). Xtail uses this value as an estimate of the *P* value for a positive (up-regulation) differential translation. Otherwise, if the translational fold change tended to be smaller than 1 (probability larger than 0.5), the posterior probability of the fold change being 1 or even larger was used to estimate the *P* value for a negative (down-regulation) differential translation. This is technically not the definition of *P* value, but as suggested in literature^58^, it is approximately equal to the classical *P* value. Therefore, for a two-tailed test, the *P* value of differential translation is estimated to be twice the summation of the elements in the upper or lower triangle, whichever is smaller, of the matrix (Fig. 1c).


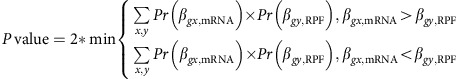


Last, the Benjamini–Hochberg multiple testing adjustment for *P* values is used at the gene level to control for false discoveries. Furthermore, from the joint probability matrix, a new probability distribution for the fold change of translation (as shown in Fig. 1c, Δ*β*_*g*_=log2FC(RPF)–log2FC(mRNA)) is generated by taking the summation of the elements in each diagonal that is parallel to the main diagonal.


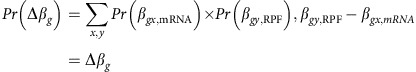


On the basis of this distribution, the credible interval of the translational fold change, [Δ*β*_*g*1_, Δ*β*_*g*2_], is then derived so that the probability of being above the upper bound is the same as the probability of being below the lower bound (equal-tailed). The credible level (*γ*) is set to be 0.95 by default and is adjustable as an optional input of Xtail.


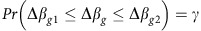


This strategy generally works very well, except for the genes that are subjected to strong transcriptional dysregulation, that is, from an extremely low-expression level in one condition to a relatively high expression in the other condition. In such cases, comparison of the mRNA or RPF between the two conditions would yield large log2FC values, which are highly sensitive to the lower expression level of mRNA or RPF in one of the two conditions. In practice, as smaller read counts generally bear more technical noise, the precision of the estimated log2FC values for such genes is usually sacrificed. This is not a critical issue for differential expression analysis with RNA sequencing data, as the absolute values of log2FC, when they are relatively large, are not critically important as long as the *P* values are reliably estimated. However, for differential translation analysis with ribosome profiling data, we need to quantitatively compare two log2FC values, of mRNA and RPF. Deviations of the two log2FC values due to technical noise of the small read counts would generate large bias towards false discoveries of uncoordinated RPF and mRNA changes, that is, falsely discovered differential translations. To address this problem, we introduced a simple but effective procedure. In addition to the comparison of log2FC distributions of mRNA and RPF, a second analysis pipeline was implemented in Xtail to compare the log2 ratios of RPF to mRNA (log2R) between the two conditions. As discussed above, theoretically this equally assesses the coordination of mRNA and RPF changes across two conditions. Specifically, following the same procedure as described above for the comparison of log2FC, Xtail generates another joint probability matrix by multiplying the two probability distributions of log2R in the two conditions (Fig. 1c). Another probability distribution for the fold change of translation, which is represented by the difference of log2R between two conditions (Δ*β*_*g*_=log2R(C2)–log2R(C1)), was derived from this joint probability matrix.


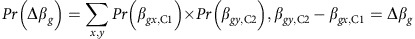


The *P* value for differential translation is calculated by taking the summation of the upper or lower triangle of the joint probability matrix, multiplied by two.


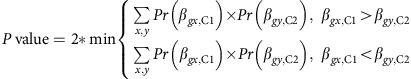


The point estimate and credible interval of the translational fold change are derived from the probability distribution of the difference of log2R between two conditions.

As introduced above, the differential translation of each gene is evaluated by these two parallel pipelines, generating two sets of results, each of which includes the *P* value, point estimate and credible interval of the differential translation. For most genes with a fairly good number of read counts and thus reliable estimates of mRNA and RPF abundances, these two pipelines yielded similar assessments of differential translation. This supports the rationality of Xtail's parallel-pipeline design. In other cases, the two sets of results can be largely different, and the more conserved one (with larger *P* value) was selected as the final assessment of differential translation.

### Evaluating performances of methods with published data

To evaluate the performances of Xtail and the other existing methods, we selected two published ribosome profiling datasets. One of them is from human prostate cancer cell PC3 after mTOR signalling inhibition with PP242 (GSE35469 in GEO)^5^, and the other one is from human primary macrophages on IFN-γ treatment (GSE66810)^14^. In both datasets, each condition includes two biological replicates. Raw read counts were subjected to median-of-ratios normalization and then fed to all the algorithms except Babel, which used raw read counts as instructed by its manual. Default parameters were used to run Babel (0.2–6), RiboDiff and baySeq. For the method of TE, genes with similar expression levels were first grouped into bins (300 genes/bin), and in each bin, the fold changes of TEs were transformed into *Z*-scores after fitting the data of these 300 genes into a normal distribution^49^. *P* values were also inferred from these distributions.

### Evaluating performances of methods with simulated data

Simulation data was generated from a published ribosome profiling dataset with four biological replicates (GSE62134) in a study of the translational responses of mouse B-cells to HuR knock-out and LPS treatment^59^. Hierarchical clustering and principle component analysis (PCA) analysis were performed to ensure no significant batch effect or obvious biological difference within the control group. To generate a theoretically all-negative comparison, two of the four replicates in the control group were randomly selected and used as condition 1, and the other two as condition 2. We then randomly selected a small portion of the genes (10–20%) and assigned fold changes larger than 1.5 to their RPF or mRNA counts in one of the two conditions. The fold changes were sampled from a gamma distribution with a shape parameter 0.6 and a scale parameter 0.5. True positives of differential translations were therefore predefined as genes of which the RPF and mRNA read counts were subjected to different levels of artificial fold changes. True negatives were predefined as genes subjected to no fold change at all or to the same fold changes on RPF and mRNA (Supplementary Data 2). Finally, this process generates a simulated ribosome profiling dataset with two samples in each condition.

A dataset without biological replication was simulated by randomly assigning one of the four control samples to condition 1 and another one for condition 2. Next, the same procedure as described above was followed to define true positives and negatives of differential translations in this single-sample data (Supplementary Data 2).

As described above, this strategy of data simulation requires data from *2n* biological replicates for a simulated dataset with *n* samples in each condition. Therefore, to generate datasets with more than two samples in each condition, we took the original data of four biological replicates, and derived a normal distribution from the four read-outs of mRNA or RPF, for each gene. Next, two additional values of mRNA or RPF expression were generated by sampling from the normal distribution above. This adds two pseudo replicates to the original four-replicate data. A three-sample dataset was then generated by assigning three replicates as condition 1 and the other three as condition 2, followed by assignment of fold changes to mRNA or RPF data in one of these two conditions.

Xtail and other existing methods were applied on these simulated datasets. The overall performances of the tested methods were assessed by ROC and precision analysis using the ROCR package.

### GO and KEGG enrichment

From the results of different methods including Xtail, Babel, RiboDiff, baySeq and TE, genes were ranked by their *P* values of differential translation, as reported by each method. The same number of top-ranked genes were selected and divided into two sets according to the direction of translational regulation, up or down. GO and KEGG enrichment analysis were conducted with these gene sets using the Database for Annotation, Visualization and Integrated Discovery^60^.

### Availability

The ready-to-use Xtail pipeline is freely available as an R package at https://github.com/xryanglab/xtail. All the datasets used in this paper, including simulated data, normalized PC3 and macrophage datasets are presented in Supplementary Data 2, 3, 4, 5, 6. A python script for counting RPF reads is provided in Supplementary Data 1.

## Additional information

**How to cite this article:** Xiao, Z. *et al*. Genome-wide assessment of differential translations with ribosome profiling data. *Nat. Commun.* 7:11194 doi: 10.1038/ncomms11194 (2016).

## Supplementary Material

Supplementary Figures Supplementary Figures 1-7

Supplementary Data 1 A custom script used to count RPF reads.

Supplementary Data 2 Simulation dataset and results.

Supplementary Data 3 PC3 dataset and results.

Supplementary Data 4 Macrophage dataset and results.

Supplementary Data 5 Sample shuffled PC3 dataset and results.

Supplementary Data 6 Sample shuffled macrophage dataset and results.

Supplementary Data 7 Top 20 genes identified by Xtail with the PC3 dataset.
