# Supplementary material for: Genome-wide assessment of differential translations with ribosome profiling data
Source: Nat Commun. 2016 Apr 4;7:11194. doi: 10.1038/ncomms11194 (PMC4822032; doi:10.1038/ncomms11194)
Supplement: Supplementary Figures — 1-7 [file ncomms11194-s1.pdf]

Supplementary Figure 1

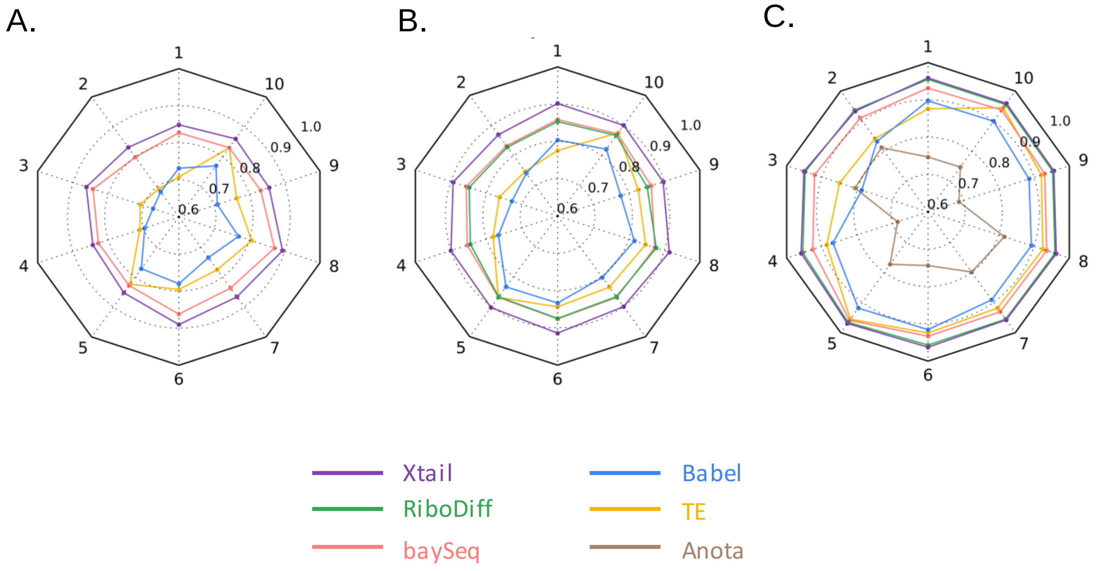

Supplementary Figure 1. Radar plots of areas under the ROC curves (AUC) from the results of different methods applied on 10 simulation datasets.

Xtail, and other existing methods were applied on 10 simulation datasets with 1 (A), 2 (B), or 3 (C) samples in each condition. For each method, the areas under the ROC curves (AUC) from the results of these 10 runs were summarized as a radar plot, in which each radius represents one of the 10 runs.

Supplementary Figure 2

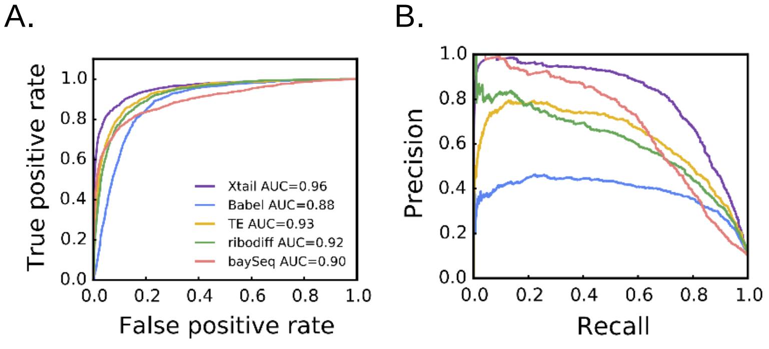

Supplementary Figure 2. The performances of different methods in differentiating translational regulations from transcriptional regulations.

Xtail and other existing methods were applied on a 2-sample simulation dataset, in which some genes were subjected to the same fold changes on mRNA and RPF (transcriptional regulation only) and some other genes subjected to different levels of fold changes on mRNA and RPF (coexisting translational and transcriptional regulations). ROC (A) and precision recall (B) curves were generated to compare the performances of different methods in differentiating translational regulations from transcriptional regulations.

Supplementary Figure 3

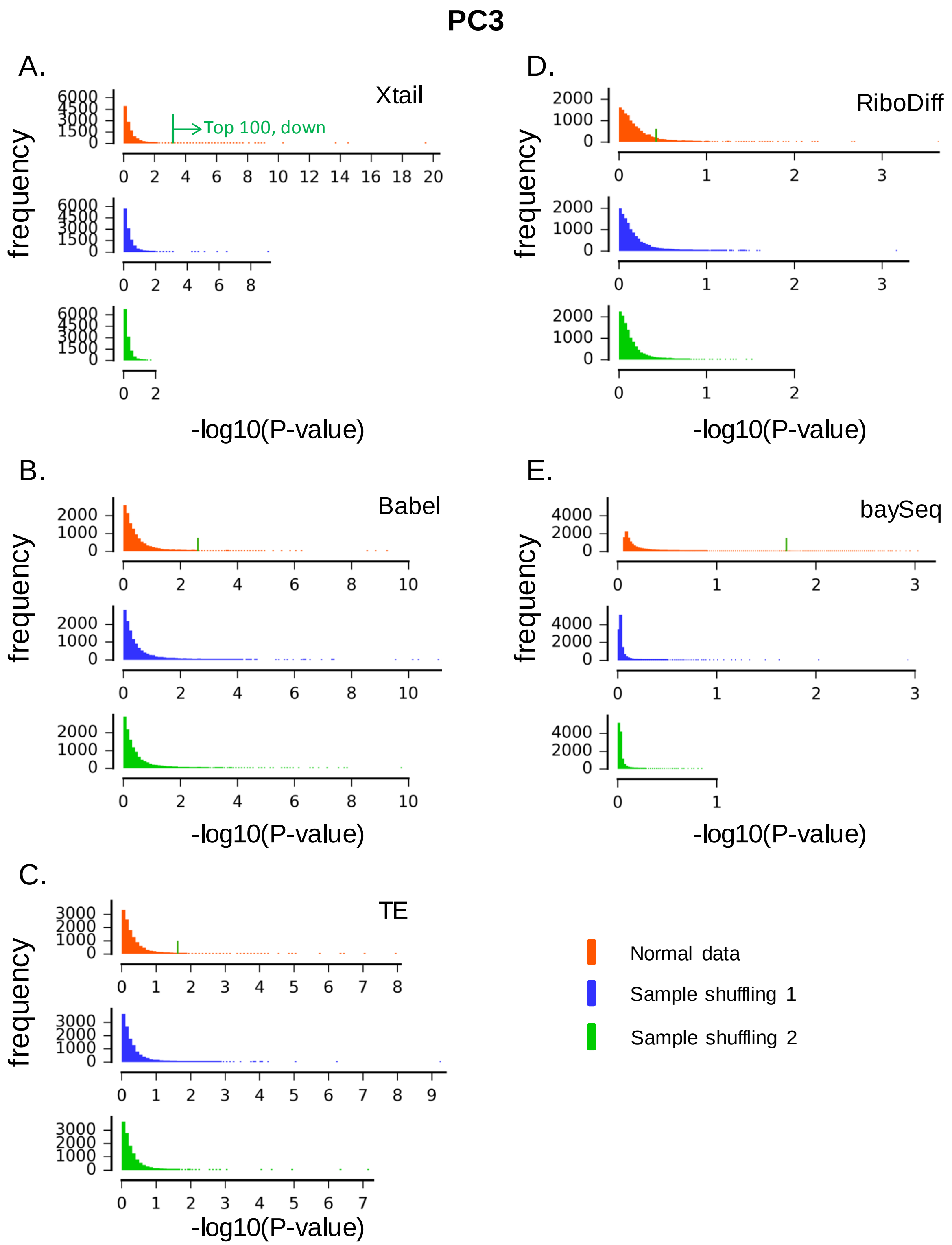

Supplementary Figure 3. Distributions of P-values from Xtail and other methods applied on the PC3 data.

Xtail and other existing methods were applied on normal and condition permutated ribosome profiling datasets from the PC3 study. P-values of all genes were collected from the results of Xtail (A), Babel (B), TE (C), RiboDiff (D), and baySeq (E). For each method, the P-value cutoff used for selection of the top 100 translationally down-regulated genes are marked on the P-value distribution.

Supplementary Figure 4

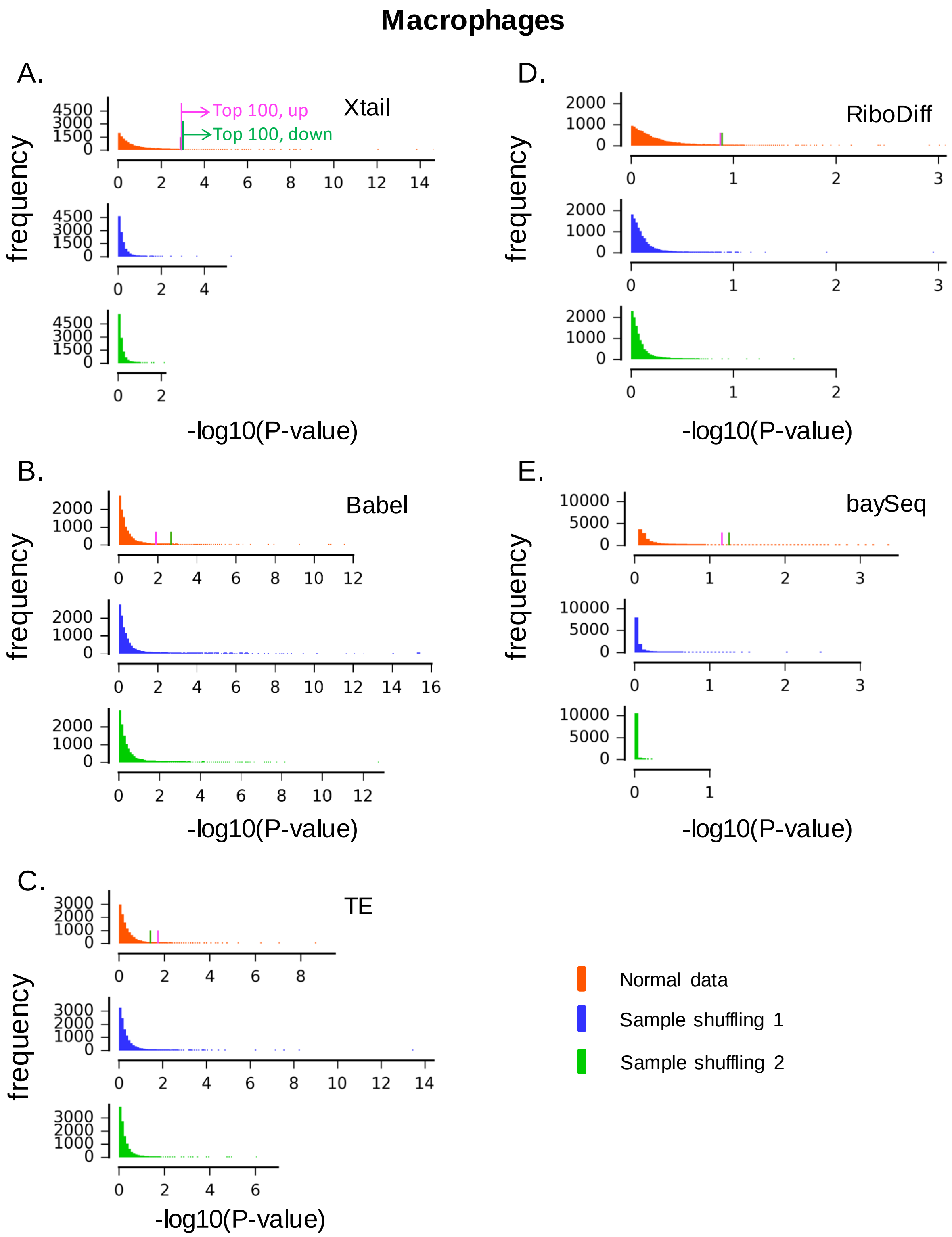

Supplementary Figure 4. Distributions of P-values from Xtail and other methods applied on the macrophage data.

Xtail and other existing methods were applied on normal and condition permutated ribosome profiling datasets from the macrophage study. P-values of all genes were collected from the results of Xtail (A), Babel (B), TE (C), RiboDiff (D), and baySeq (E). For each method, the P-value cutoffs used for selection of the top 100 translationally up- and down-regulated genes are marked on the P-value distributions.

Supplementary Figure 5

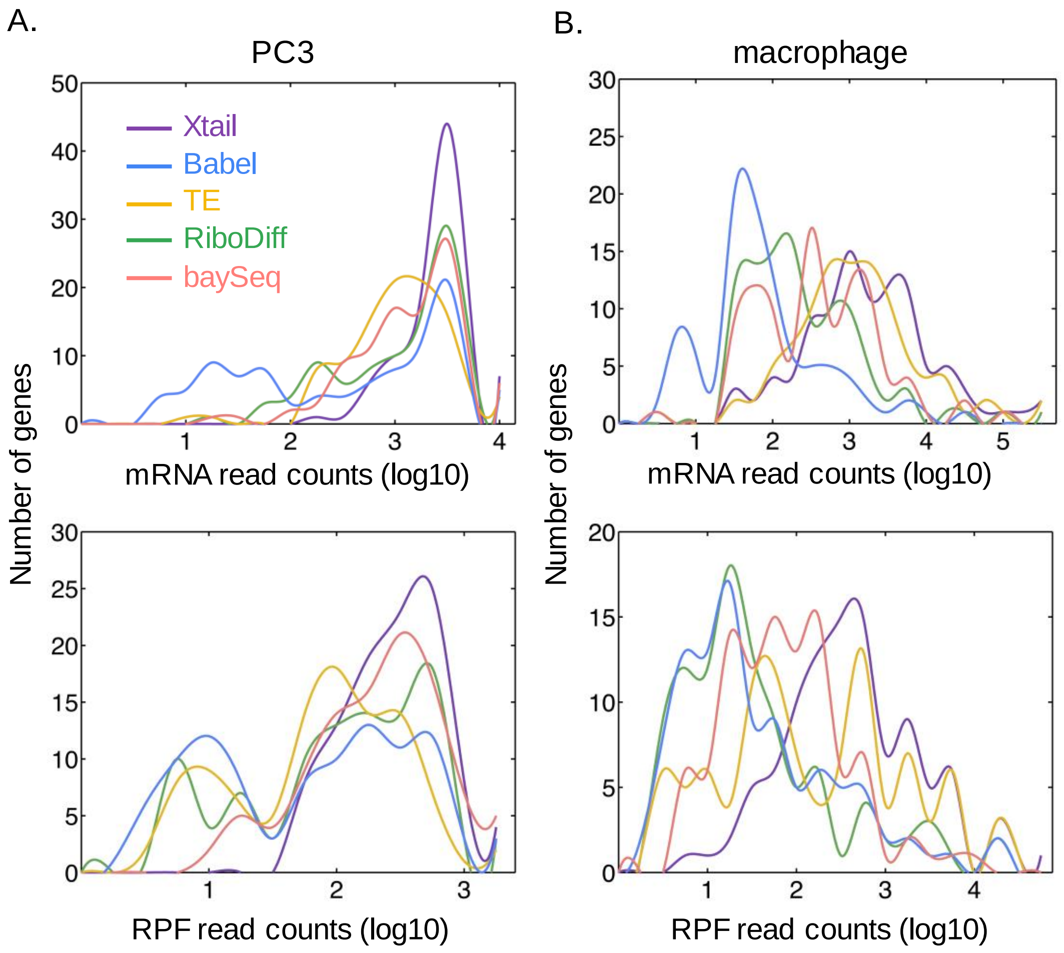

Supplementary Figure 5. Expression distributions of the top-100 differentially translated genes identified by different methods.

Different methods were applied to identify the top-100 differentially translated genes in the PC3 and macrophage studies. For each method, distributions of the mRNA and RPF counts of these 100 genes were generated for the PC3 (A) and macrophage (B) studies.

Supplementary Figure 6

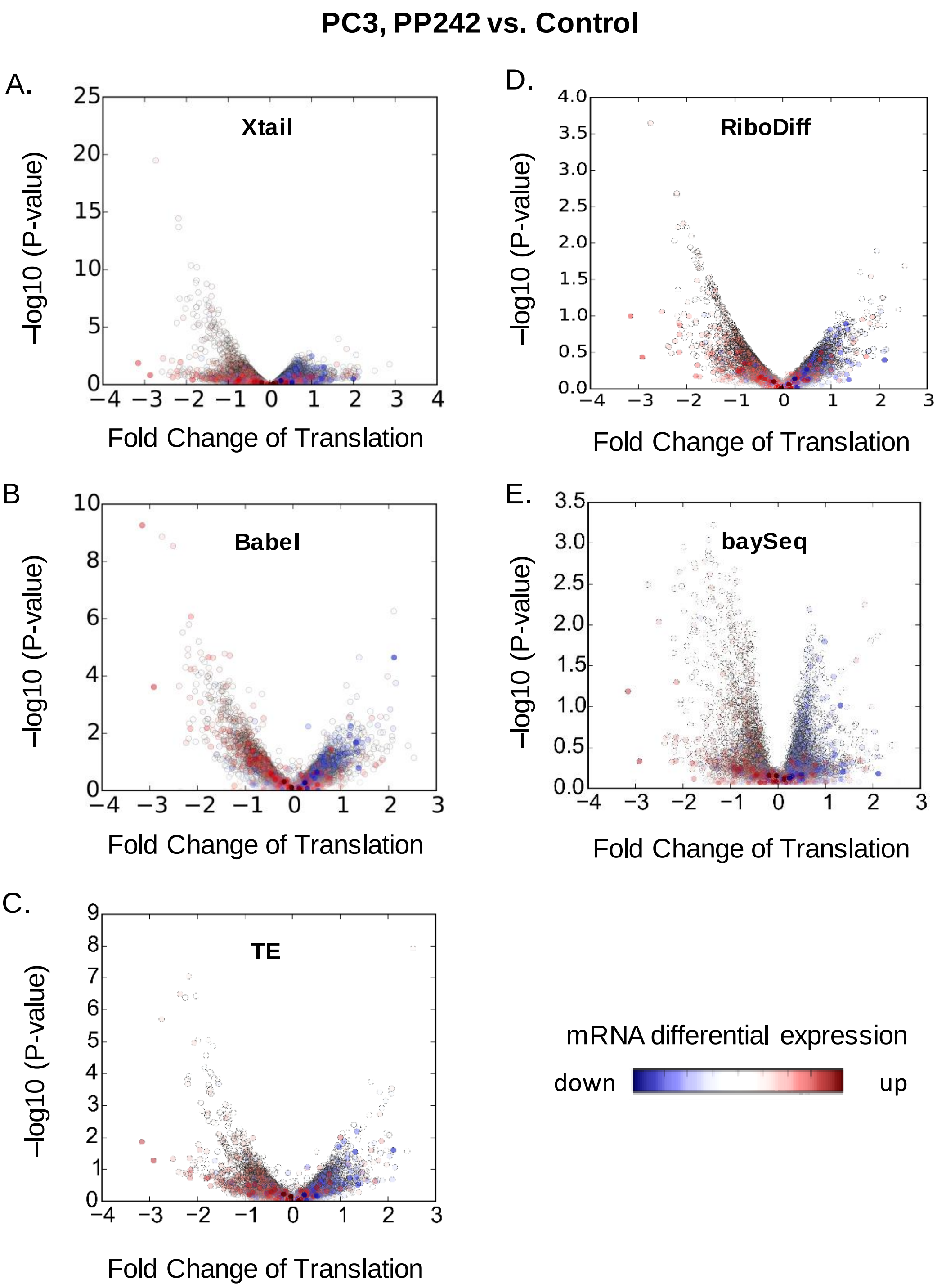

Supplementary Figure 6. Volcano plots of the results from different methods applied on the PC3 data.

Xtail and other existing methods were applied on the ribosome profiling dataset from the PC3 study. The results were summarized as volcano plots for Xtail (A), Babel (B), TE (C), RiboDiff (D), and baySeq (E). Each dot, representing a particular gene, was color-coded by P-value (–log10) of its mRNA differential expression across two conditions, which was obtained with DEseq2.

Supplementary Figure 7

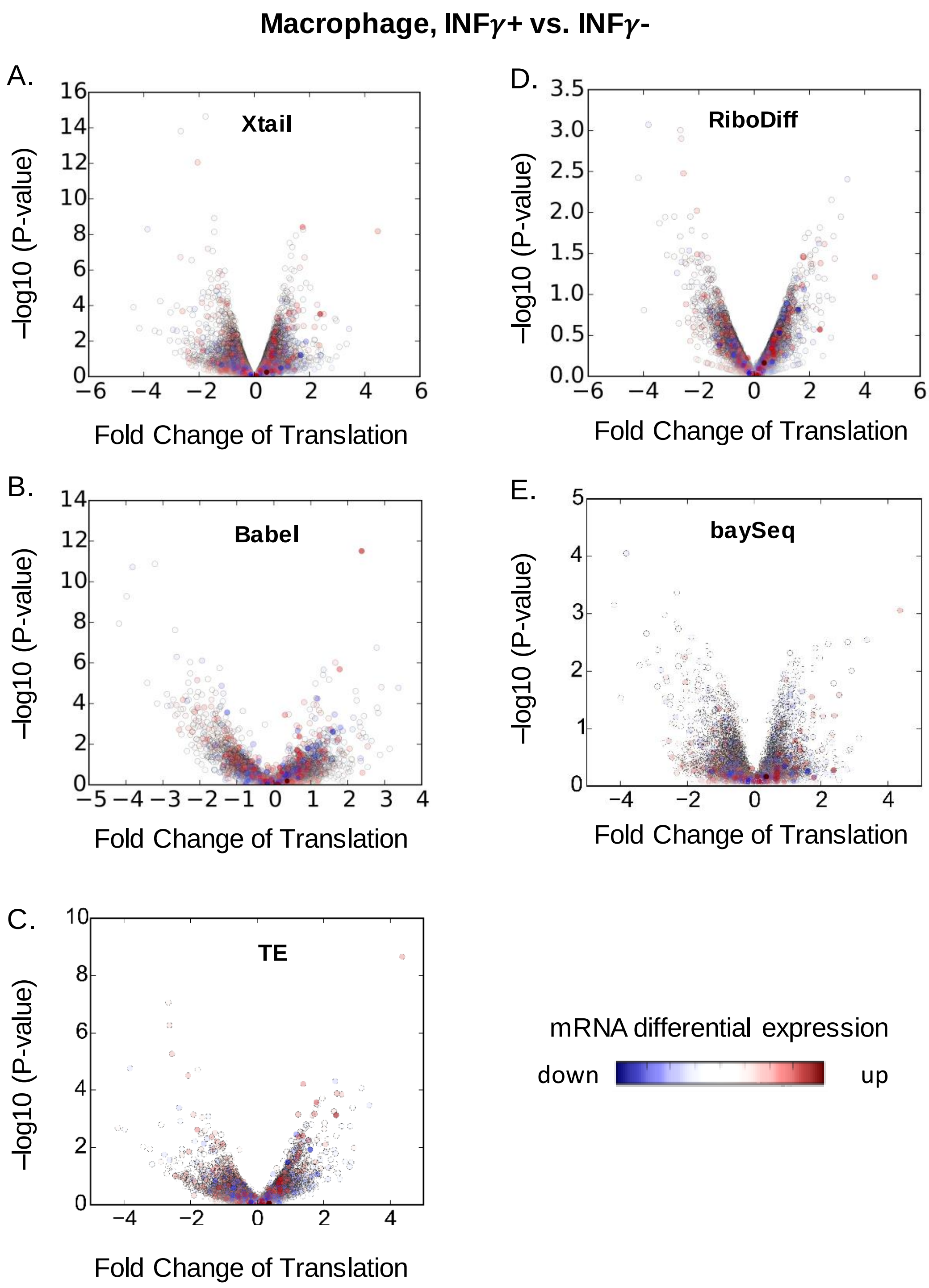

Supplementary Figure 7. Volcano plots of the results from different methods applied on the macrophage data.

Xtail and other existing methods were applied on the ribosome profiling dataset from the macrophage study. The results were summarized as volcano plots for Xtail (A), Babel (B), TE (C), RiboDiff (D), and baySeq (E). Each dot, representing a particular gene, was color-coded by P-value (–log10) of its mRNA differential expression across two conditions, which was obtained with DEseq2.
